# Environmental distribution of *Echinococcus*- and *Taenia* spp.-contaminated dog feces in Kyrgyzstan

**DOI:** 10.1017/S003118202300118X

**Published:** 2024-01

**Authors:** Kubanychbek K. Abdykerimov, Philipp A. Kronenberg, Myktybek Isaev, Giulia Paternoster, Peter Deplazes, Paul R. Torgerson

**Affiliations:** 1 Section of Epidemiology, Vetsuisse Faculty, University of Zurich, Zurich, Switzerland; 2 Kyrgyz Research Institute of Veterinary named after A. Duisheev, Ministry of Education and Science of the Kyrgyz Republic, Bishkek, Kyrgyzstan; 3 Vetsuisse and Medical Faculty, Institute of Parasitology, University of Zurich, Zurich, Switzerland; 4 Microbiology and Molecular Biology, Institute of Chemistry and Biotechnology, Zurich University of Applied Sciences (ZHAW), Wädenswil, Switzerland; 5 Federal Food Safety and Veterinary Office FSVO, Bern, Switzerland; 6 Department of Gastroenterology and Hepatology, University Hospital Zurich, Zurich, Switzerland

**Keywords:** dog feces, *Echinococcus granulosus* s.l, *Echinococcus multilocularis*, environmental contamination, McMaster method, *Taenia crassiceps*, *Taenia hydatigena*, *Taenia ovis*

## Abstract

Recently, there have been epidemics of human cystic echinococcosis (CE) and alveolar echinococcosis (AE) in Kyrgyzstan. This study investigated 2 districts for the presence of *Echinococcus granulosus* s.l. and *Echinococcus multilocularis* eggs; species identity was confirmed by polymerase chain reaction in dog feces and the level of environmental contamination with parasite eggs in 2017–2018 was also investigated. In the Alay district 5 villages with a high reported annual incidence of AE of 162 cases per 100 000 and 5 villages in the Kochkor district which had a much lower incidence of 21 cases per 100 000 were investigated. However, the proportion of dog feces containing *E. granulosus* s.l. eggs was ~4.2 and ~3.5% in Alay and Kochkor respectively. For *E. multilocularis*, the corresponding proportions were 2.8 and 3.2%. Environmental contamination of *Echinococcus* spp. eggs was estimated using the McMaster technique for fecal egg counts, weight and density of canine feces. The level of environmental contamination with *E. multilocularis* eggs was similar at 4.4 and 5.0 eggs per m^2^ in Alay and Kochkor respectively. The corresponding values for *E. granulosus* s.l. were 8.3 and 7.5 eggs per m^2^. There was no association between village or district level incidence of human AE or CE and the proportion of dog feces containing eggs of *Echinococcus* spp. or the level of environmental contamination. Increased contamination of taeniid eggs occured in the autumn, after the return of farmers with dogs from summer mountain pastures.

## Graphical abstract

**Figure:**
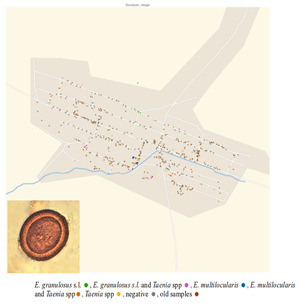

## Introduction

Alveolar echinococcosis (AE) and cystic echinococcosis (CE) are major zoonoses in central Asia. AE is caused by the parasite *Echinococcus multilocularis* whilst CE is caused by *Echinococcus granulosus* s.l. Both diseases cause extensive human morbidity, and AE is usually fatal if left untreated (Torgerson *et al.*, 2008). In Kyrgyzstan, there is now evidence for a major epidemic of AE (Usubalieva *et al.*, 2013; Raimkylov *et al.*, 2015; Counotte *et al.*, 2016; Paternoster *et al.*, 2020). The annual burden of echinococcosis in Kyrgyzstan for 2013 was estimated to be 11 915 [95% uncertainty interval (UI): 4705–27 114] disability-adjusted life years (DALYs) for AE and 3052 [95% UI: 1508–5205] DALYs for CE (Counotte *et al.*, 2016).

Foxes (*Vulpes vulpes*) are one of the usual definitive hosts of *E. multilocularis*. Studies on foxes in Naryn district in Kyrgyzstan have demonstrated a prevalence of *E. multilocularis* infection of 64% (Ziadinov *et al.*, 2010). Dogs have been shown to be suitable definitive hosts for *E. multilocularis* with similar egg reproduction to foxes (Kapel *et al.*, 2006). In dogs in Naryn district, the prevalence of *E. granulosus* s.l. has previously been reported to be ~18%, whilst that of *E. multilocularis* is ~19% (Ziadinov *et al.*, 2008). *Echinococcus* spp. have complex life cycles where carnivore hosts play an important role in the transmission in Kyrgyzstan. It is hypothesized that in Kyrgyzstan dogs play an important role in the zoonotic risk; however, the wild animal cycle including foxes functions as a reservoir for *E. multilocularis* (Ziadinov *et al.*, 2008, 2010; Mastin *et al.*, 2015; Van Kesteren *et al.*, 2017).

It has been hypothesized that an increase in human CE and AE in central Asia may be related to the dissolution of the Soviet Union in 1991 with fundamental sociocultural, economic and structural changes (Torgerson, 2013). Furthermore, a decline in the activities of veterinary services is reported, including a decrease or absence of veterinary control over meat production. The process of domestic slaughter has increased, as well as the privatization of large livestock farms. A further consequence of the crisis has been increasing human poverty (Torgerson, 2013) along with an increase in the number of dogs in both rural and urban areas (Torgerson *et al.*, 2006).

The first case of human AE in Kyrgyzstan was diagnosed in 1996, but until 2003 only small numbers of cases were registered annually. The number of reported cases has increased significantly since 2004 (Usubalieva *et al.*, 2013). Human AE has a prolonged latent period. The epidemic of human AE started ~15 years after the dissolution of the Soviet Union. Therefore, it is hypothesized that the socioeconomic changes that lead to the increase in incidence of CE also created the conditions that allowed the colonization of dogs by *E. multilocularis.* This has resulted in an onward transmission to humans due to the close contact between dogs and humans and hence an epidemic of human AE (Torgerson, 2013).

In Kyrgyzstan, the national annual incidence of reported human cases of CE 25 years ago was ~4 cases per 100 000. In recent years it is ~15 per 100 000. Of serious concern is that the much more pathogenic form of AE has emerged as a major public health problem. During the last 20 years AE has increased to ~200 reported cases per year (Usubalieva *et al.*, 2013; Raimkylov *et al.*, 2015; Counotte *et al.*, 2016; Paternoster *et al.*, 2020). In certain districts, very high prevalences of human AE have been detected by ultrasound surveillance. For example, an ultrasound study in the south of Kyrgyzstan (Bebezov *et al.*, 2018) documented a prevalence of AE of 4.2%.

The aim of this study is to investigate the environmental contamination of taeniid eggs in high and lower incidence areas for AE. We also wished to investigate seasonal associations which may give indications of transmission and if control could be targeted. We also hypothesized if there may be a link between the contamination with eggs of *E. multilocularis* in public areas and the local incidence of human AE and if dogs are the main source of infection for the human population.

## Materials and methods

### Study area and sample collection

**Figure 1.**
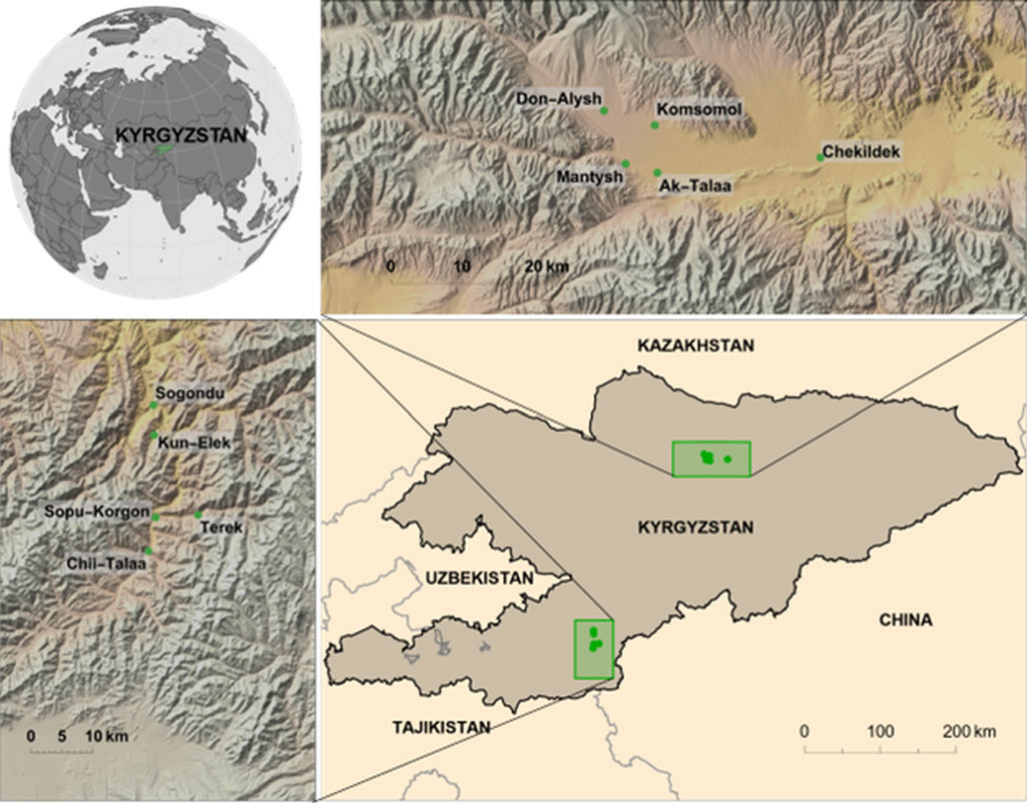
Location of the expedition points in Kyrgyzstan and separately on top of the Kochkor (low AE incidence) district and on the left side of the Alay (high AE incidence) district.

For the study, villages were selected based on human incidence data (Paternoster *et al.*, 2020). Five villages were selected in the Alay district where the reported incidence of AE between 2014 and 2016 was amongst the highest in Kyrgyzstan. For comparison 5 villages were selected in the Kochkor district which has a substantially lower reported incidence of human AE. Terek, Sopu-Korgon, Sogondu, Kun-Elek and Chii-Talaa villages were explored in Alay district; Mantysh, Komsomol, Don-Alysh, Chekildek and Ak-Talaa villages in Kochkor district (Fig. 1). For each village, samples of all fresh dog feces were collected and assigned a specific identification number. Fresh feces were intact feces that had the appearance of being recently deposited. For older fecal samples, which were dried out with some evidence of degradation, the number and position were recorded. Dog feces were identified by morphology, size and content. The GPS coordinates of all observed fecal samples (fresh and old) were recorded. It was only possible to access public parts of the villages for sample collection and not, for example, enclosed private gardens. The area of the parts of the village that was accessible, and samples taken was estimated using the Google Maps Area Calculator Tool (Frančula, 2020).

All samples were frozen for 5 days at minus 80°C prior to analysis for safety reasons.

### Mapping of surgical cases

The national surveillance system for surgical cases of human echinococcosis compiled by the State Sanitary and Epidemiological Service in Bishkek, Kyrgyzstan was used. These data have been previously reported by Paternoster *et al.* (2020). For this study the community level incidence of AE was used to explore if there was any association with the level of *Echinococcus* spp. contamination in dog feces and density of environmental contamination and with human incidence was used.

### Egg isolation

The eggs were isolated according to a previously described study (Mathis *et al.*, 1996) with some modifications. In brief, the samples were thawed; then 2 g of feces were weighed into a 15 mL Falcon tube. Subsequently, a 1:4 solution of phosphate-buffered saline–Tween–sodium azide was added, shaken vigorously for 1 min and centrifuged for 10 min at 500***g***. After removing the supernatant, the pellet was re-suspended in 8 mL of zinc chloride solution (density 1.45), then the samples were shaken again and centrifuged for 30 min at 400***g***. Afterwards, the supernatant was passed through nylon meshes with a mesh size of 40 and 21 *μ*m (Lanz-Anliker, Rohrbach, Switzerland). A 21 *μ*m nylon mesh was used to collect the taeniid eggs. The sediment was placed in a flat-walled Nunc tube and examined for the presence of taeniid eggs using an inverted microscope. Positive samples were collected and centrifuged at 500***g*** for 5 min.

### DNA isolation from taeniid eggs

An isolation of the DNA was achieved by using a method adapted from that previously described (Trachsel *et al.*, 2007). In brief, the pellet was transferred into a 1.5 mL Eppendorf tube and centrifuged for 1 min at 8000***g***. The supernatant was removed, and the pelleted eggs were re-suspended in 200 *μ*L distilled water. Then 25 *μ*L of 1 m potassium hydroxide and 7 *μ*L dithiothreitol were added to the sample and vortexed, followed by an incubation at 65°C for 15 min.

Furthermore, 60 *μ*L 2 m Tris-hydrochloric acid (HCl) (pH 8.4) and 2 *μ*L concentrated HCl (12.4 N/>37%) were added. After the addition of 200 *μ*L of buffer AL (QIAamp DNA Mini Kit, Qiagen) and 20 *μ*L proteinase K, the sample was incubated at 56°C for 10 min. A 50 *μ*L of Chelex (50%) was added and the mix was put on a rotor at room temperature between 30 min and 3 h. The tubes were then centrifuged at 16 000***g*** for 1 min and the supernatant was transferred to a fresh tube together with 200 *μ*L pure ethanol. The whole mix was vortexed and spun down.

The samples were applied to Qiagen spin columns and centrifuged for 1 min at 8000***g***. The columns were washed with 300 *μ*L buffer AW1 and centrifuged for 1 min at 8000***g***. This washing step was repeated once again. Then the columns were washed with buffer AW2 and centrifuged for 1 min at 8000***g***. After the repetition of the washing step with AW2, the columns were centrifuged empty at full speed for 3 min. Next, the columns were placed in a 1.5 mL Eppendorf tube, 100 *μ*L AE elution buffer was added and incubated for 1 min at room temperature, to elute the DNA from the membrane. As a last step, the columns were centrifuged for 1 min at 8000***g*** and the flow-through was collected.

### Polymerase chain reaction (PCR)

For the PCR a multiplex PCR adapted from a previous protocol (Trachsel *et al.*, 2007) with a commercial kit (Qiagen Multiplex PCR Kit, Qiagen, Hilden, Germany) was used. First, a mastermix containing 25 *μ*L Qiagen Mastermix, 18 *μ*L distilled water, both from the multiplex PCR Kit, as well as 5 *μ*L primermix were prepared in PCR tubes. The primermix is composed of 80 *μ*L Cest5 primers and 10 *μ*L from Cest1, 2, 3, 4 primers as well as 380 *μ*L distilled water. Primers Cest1 (5′-TGC TGA TTT GTT AAA GTT AGT GAT C-3′) and Cest2 (5′-CAT AAA TCA ATG GAA ACA ACA ACA AG-3′) amplified a 395 bp stretch of the *nad*1 gene of *E. multilocularis*; primers Cest3 (5′-YGA YTC TTT TTA GGG GAA GGT GTG-3′) and Cest5 (5′-GCG GTG TGT ACM TGA GCT AAA C-3′) amplified a 267 bp stretch of the *rrnS* of *Taenia* spp. and primers Cest4 (5′-GTT TTT GTG TGT TAC ATT AAT AAG GGT G-3′) and Cest5 amplified a 117 bp stretch of the *rrnS* of *E. granulosus* (s.l.). Finally, 2 *μ*L of DNA solution was added. To confirm the validity of the PCR, positive as well as negative controls were implemented.

The cycling conditions were 15 min at 95°C, 30 s at 94°C, 90 s at 58°C with a repetition of 40 cycles and another 10 min at 72°C.

### Sequencing

For a more accurate determination of the taeniid species, all sequencing was performed at Microsynth Switzerland. Therefore, the DNA was purified by using a commercial kit (MinElute PCR Purification Kit, Qiagen) and sent for sequencing.

### Density of contamination

Estimates of the fecal egg counts were performed using the McMaster technique (Deplazes and Eckert, 1988*a*). For estimations of the mean fecal egg count only samples with mono-infections with taeniids, with *E. granulosus* s.l., *E. multilocularis* or *Taenia* spp. (species not determined), respectively, were used. These infections were diagnosed by PCR as described above.

To calculate the average mass of a canine fecal sample, 50 samples were collected, randomly weighed and the mean weight was calculated. Total numbers of eggs were the eggs per g multiplied by the average fecal weight. Density of contamination was estimated according to:1
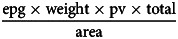
where epg = mean eggs per g of *E. granulosus* s.l., *E. multilocularis* or *Taenia* spp. eggs; weight = mean weight of a single fecal sample; pv = proportion of fresh fecal samples containing *Echinococcus* spp. or *Taenia* spp. (including mixed infections); total = total number of fecal samples found (fresh and old) and area = sample area from which feces were searched and collected.

### Statistical analysis

For the statistical evaluation the statistic program R (R Core Team, 2022) was used. Poisson regression was used to examine if there was any association between the prevalence of *Echinococcus* spp. in the canine fecal samples or the level of contamination with parasite eggs and the incidence of echinococcosis from the national surveillance reports.

For egg contamination per m^2^, the fecal egg counts were analysed using the eggCounts package in R (Torgerson *et al.*, 2014). Uncertainty in the total egg contamination was estimated by using equation (1). Here, the uncertainty in the proportion of fecal samples containing parasite eggs was estimated by using 2000 beta random variables from Beta (*a* + 1, *b* + 1) where *a* is the number of fresh fecal samples positive for *Echinococcus* spp. or *Taenia* spp. and *b* is the number of fresh fecal samples that were negative. Uncertainty in the fecal egg counts was calculated by using 2000 draws from the posterior distribution from the single sample egg count function in eggCounts. Each of these random draws was analysed in equation (1). The 2.5, 50 and 97.5% quantiles of the distribution of the 2000 resulting samples were estimates of the median and 95% confidence interval (CI) limits of the fecal egg contamination in terms of eggs per m^2^. All data and R code are provided as Supplementary files 1 and 2.

## Results

### Taeniid egg contamination in fecal samples of dogs

A total of 2013 fresh samples of dog feces was examined. In 53 samples *E. multilocularis* was detected (of which 10 were mixed infections with *Taenia* spp.), 54 samples with *E. granulosus* s.l. (of which 27 were mixed infections with *Taenia* spp.) and 285 samples positive for *Taenia* spp. (of which 37 were mixed infections with *Echinococcus* spp.). Results of all samples are summarized in Table 1.

**Table 1.** Overview of the number of positive taeniid samples in dog feces (2017–2018)

Name of village	Total old fecal samples	Total fresh fecal samples	*Echinococcus multilocularis*	*Echinococcus granulosus* s.l.	*Taenia* spp.
Terek	494	183	8	6	29
Sopu-Korgon	589	184	0	11	59
Sogondu	811	193	5	12	33
Kun-Elek	384	204	0	9	20
Chii-Talaa	611	210	13	3	24
Mantysh	764	197	3	7	20
Komsomol	963	241	14	13	53
Don-Alysh	887	207	6	9	37
Chekildek	725	195	8	9	32
Ak-Talaa	837	199	0	0	12

A total of 7065 old samples of dog feces were detected and recorded. The minimum number of old samples of dog feces found was in February in the Alay district, which can be explained by the large amount of snow that fell during that period. Also, the minimum number of old samples of dog feces found was in the Alay district in June, since at that time more dogs were with farmers on summer pastures in the mountains. The maximum number of old samples of dog feces in the Alay district was found in September and November.

In the Kochkor district, fewer old dog fecal samples were found in June than in other months (September, November and February), as at that moment more dogs were on summer pastures in the mountains with farmers.

**Figure 2.**
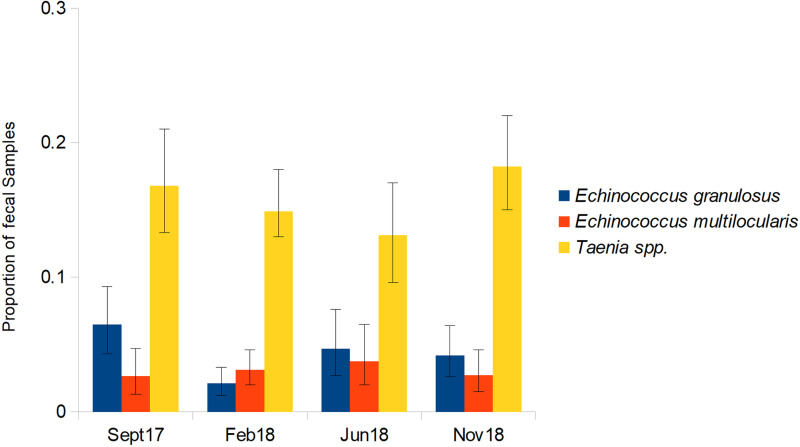
Proportion of fecal samples (±95% confidence intervals) containing *Echinococcus granulosus* s.l., *Echinococcus multilocularis* and *Taenia* spp. eggs across the 4 sampling periods.

After PCR testing of dog fecal samples, we selected 298 samples for the quantitative egg counts by the McMaster method, 26 samples with *E. granulosus* s.l., 42 with *E. multilocularis* and 230 with *Taenia* spp. The mean eggs per g were 1607 (CI 1188–2144) for *E. granulosus* s.l.; 1287 (CI 1009–1623) for *E. multilocularis* and 1199 (CI 1092–1314) for *Taenia* spp. There was little variation in the proportion of samples in which *Taenia* spp. and *Echinococcus* spp. eggs were found across the 4 sampling periods. Only between September 2017 and February 2018 an apparent decrease in the proportion of samples containing *E. granulosus* s.l. eggs (*P* < 0.01, Fig. 2) was observed.

**Figure 3.**
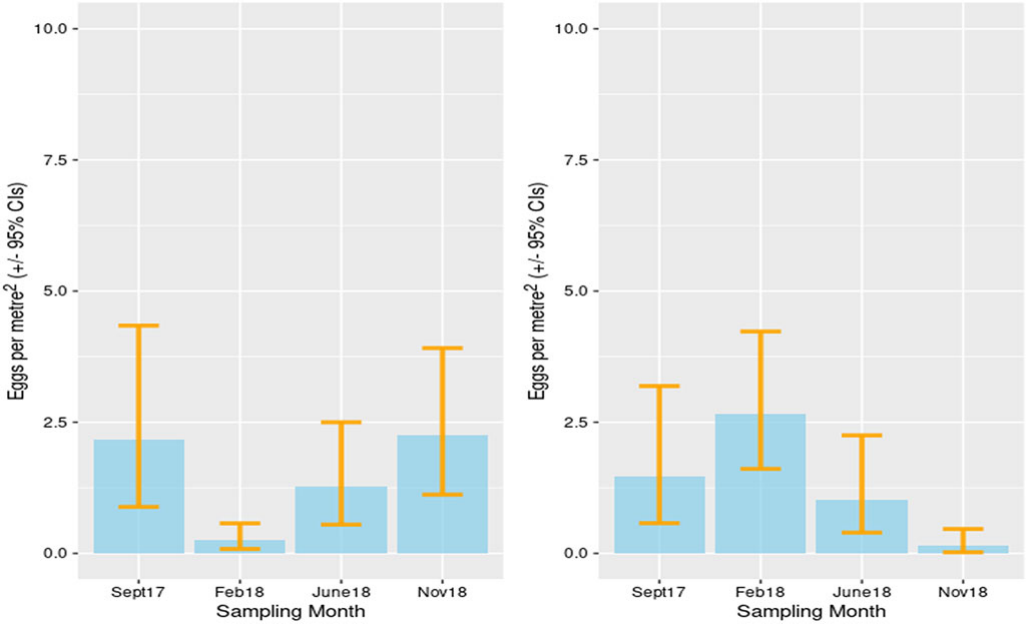
Estimated contamination of *E. multilocularis* eggs in canine feces: Alay district (left) and Kochkor district (right).

There was some heterogeneity in the level of contamination of eggs of *E. multilocularis*, which ranged from 17 eggs per m^2^ (CI 8.6–31) for the village of Chekildek in Kochkor district, to several villages having no *E. multilocularis* detected. There were no significant differences between the levels of contamination in villages in Kochkor district compared to villages in Alay district. There was some evidence of decreased contamination of the environment in the winter months in the Alay district, whereas in the Kochkor district there was some evidence of increased levels of contamination (Fig. 3).

**Figure 4.**
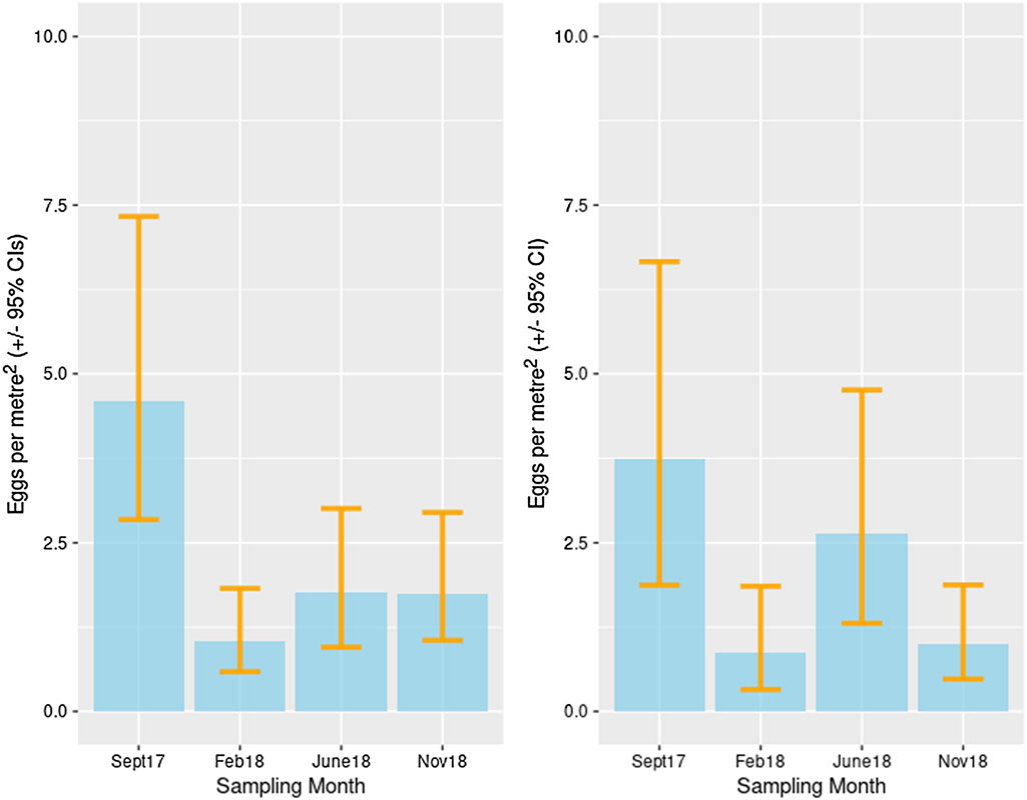
Estimated contamination of *E. granulosus* s.l. eggs in canine feces: Alay district (left) and Kochkor district (right).

For *E. granulosus* s.l. there was also considerable heterogeneity with the level of contamination ranging from 21 eggs per m^2^ (CI 9.9–40.5) for the village of Chekildek in the Kochkor district to 2.9 eggs per m^2^ (CI 0.9–7.2) in Chii-Talaa in the Alay district (Fig. 4). There was some evidence of lower contamination rates in both districts during the winter (Fig. 4).

**Figure 5.**
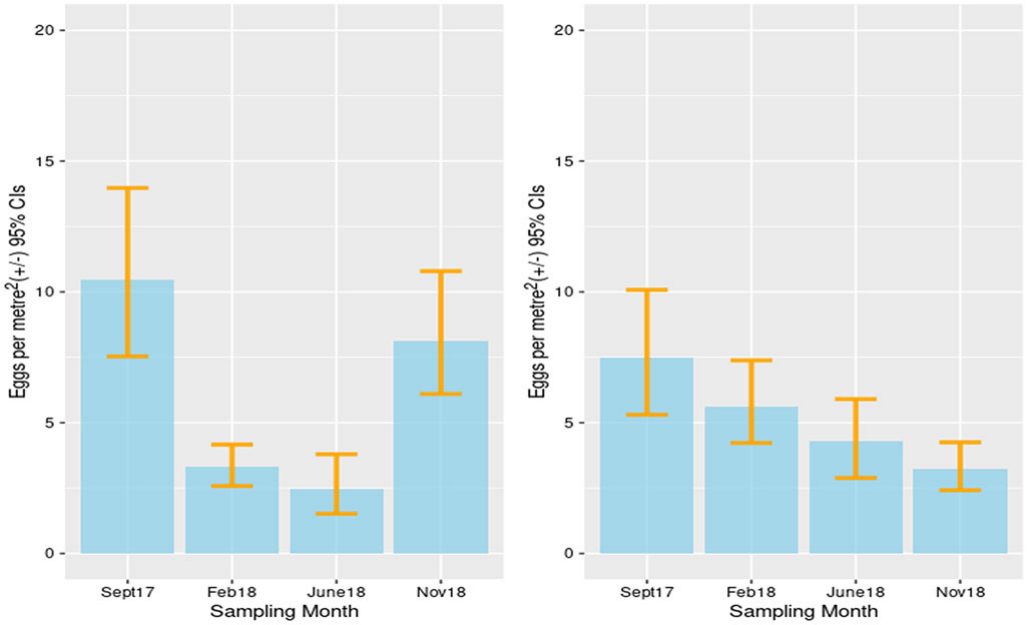
Estimated contamination of *Taenia* spp. eggs in canine feces: Alay district (left) and Kochkor district (right).

Contamination with *Taenia* spp. eggs decreased from the autumn throughout the winter before increasing again the following autumn in the Alay district (Fig. 5). In Kochkor district, there was a continuous decrease during the period of this study.

There was no association with the level of environmental contamination in public areas, and the reported village level incidence reported in the national surveillance system. There was no evidence for a difference in the proportion of dog feces containing *E. multilocularis* eggs in the high incidence district of Alay in comparison to the lower incidence district of Kochkor. The same is true in respect of the level of contamination. There was also no evidence of an association between the proportions of dog fecal samples containing *E. granulosus* s.l. eggs and the incidence of CE in the human population (*P* > 0.05) and the same is true in respect of the level of contamination. Summary data together with the proportion of dog fecal samples containing *Echinococcus* spp. parasite eggs isolated from dog feces and the level of contamination are given in Table 2.

**Table 2.** Proportion of fecal samples containing *Echinococcus* spp. eggs and the level of contamination in public areas of the high and low incidence districts

District	Incidence AE^a^	Incidence CE^a^	Proportion (%) of samples positive for *E. multilocularis*^b^	Cumulative contamination, *E. multilocularis*^c^	Proportion (%) of samples positive for *E. granulosus* s.l.^b^	Cumulative contamination, *E. granulosus* s.l.^c^
Alay	162	42	2.8 (1.8–4.0)	4.4 (2.7–6.6)	4.2 (3.0–5.6)	8.3 (5.4–12.5)
Kochkor	21	25	3.2 (2.3–4.5)	5.0 (3.2–7.5)	3.5 (2.4–4.7)	7.5 (4.9–11.7)

a Annual incidence per 100 000 population.

b Proportion of canine fecal samples (%) (95% CI).

c Estimated total eggs per m^2^ from September 2017 to November 2018 (95% CI).

### Sequencing of randomly selected positive *Taenia* spp. fecal samples of dogs

For the *Taenia* species to be identified, 15 samples from each year were randomly selected and sequenced (Trachsel *et al.*, 2007). Of these 30 samples, 26 were *Taenia hydatigena*, 3 were *Taenia crassiceps* and 1 was *Taenia ovis*.

## Discussion

Widespread contamination of village public spaces with dog feces containing eggs of *E. multilocularis*, *E. granulosus* and *Taenia* spp. was demonstrated. This represents a source of infection for the human population for *Echinococcus* spp. and *T. crassiceps*. However, it was not possible to detect any association between the intensity of environmental contamination and the officially reported incidence of AE or CE. The incidence of AE in the Alay district was nearly 4 times higher compared to the Kochkor district (Table 2). Yet there was a very similar proportion of dog feces containing *E. multilocularis* eggs and similar levels of contamination. Likewise, no differences could be detected in the proportion of fecal samples with eggs of *E. granulosus* s.l. or the level of contamination. This would support the hypothesis that a direct environmental contamination of public areas is not the main driver of this epidemic of human echinococcosis in Kyrgyzstan. However, a weakness of our approach was our method for estimating the level of contamination in the villages. We were not able to access considerable parts of the villages, such as private gardens, to collect samples and check for fecal contamination. It is possible that these areas had a different intensity of contamination compared to the areas we were able to access, and this could generate bias. Arguably, there might also be greater contact between dog feces and humans in areas such as private gardens which might be a greater driver of transmission. However, it is worth noting that there appears to be quite considerable accumulations of dog feces in places where local people frequent, often such as around the village school, mosque or shops, where we were able to obtain samples (see figures in Supplementary file 3). It was also assumed that the feces observed and/or collected were dog feces based on the morphology. It is possible that some may have been feces of foxes or other wild carnivores. Ideally DNA analysis of the samples would confirm the species origin of the samples. In Tibetan plateau village communities, which have a similarly high incidence of human AE as the villages in the present study, 13 of 155 carnivore fecal samples (8.3%) were shown to be fox feces by PCR analysis. The remainder were dog feces (Vaniscotte *et al.*, 2011). However, regardless of this the estimated contamination levels would be valid.

There is also a long latent period between infection and the development of the disease. Therefore, cases reported in the national surveillance system will represent infection events of some time, possibly of years ago. Thus, the levels of contamination may have changed between when the infection event occurred and the detection of human cases through surveillance. It is also assumed that the reporting of cases to the national surveillance system is accurate and has no under-reporting in either of the 2 districts. Nearly all cases of AE and CE are treated in either Bishkek or Osh. Suitable medical facilities are not available in the rural areas where this study was undertaken. But the patient origin is reported from the hospital records, and this would suggest there is minimal bias in reporting, as nearly all reports originate from these main medical centres. In the Alay district an ultrasound surveillance study was previously undertaken (Bebezov *et al.*, 2018) and reported an AE prevalence of 4.2% in that district, which is consistent with the very high reported incidence of treated cases.

Nevertheless, our data might also point to other sources of infection. The water supply has been shown to be a risk factor for human echinococcosis and a systematic review and a meta-analysis has suggested it may represent a considerable attributable fraction for human echinococcosis (Torgerson *et al.*, 2020). Investigations into potential contamination of the water supplies of these villages are ongoing. It is noteworthy that much of the population of these villages does not have access to a safe and sanitary water supply.

There was some, albeit inconsistent evidence for seasonal variations in the contamination of village spaces. Thus, in the Alay district, the contamination levels for both *Echinococcus* species and for *Taenia* spp. declined in the winter before recovering by the following autumn. It can be hypothesized that the large number of eggs of the parasites in the autumn period of the year is due to the fact that dogs descend with farmers from summer pastures to villages before the onset of winter. Farmers on summer pastures in the mountains slaughter sheep themselves; they feed the affected organs with echinococcosis cysts (lungs and liver) to dogs. Dogs may also prey on small mammals which are a natural reservoir of *E. multilocularis* and *T. crassiceps*. Data of the results from a previous study (Abdyjaparov and Kuttubaev, 2004) indicate the presence of AE invasion in 10 species of rodents in alpine districts in Kyrgyzstan. These included the area around Sari Tash (altitude 3170 m), where *E. multilocularis* infection of 0.8% of rodents was documented. This included 4% of red marmots (*Marmota caudata*). In Sary Mogal (altitude 2980 m), which is a neighbouring settlement to Sari Tash, the study by Bebezov *et al.* (2018) documented a high prevalence of human AE. The result shows that the majority of *Taenia* infections were *T. hydatigena*, a species maintained in a domestic cycle mainly with dogs and sheep and goats, i.e. the same as *E. granulosus*; on the other hand *T. crassiceps* has a cycle comparable with *E. multilocularis*. Three of 30 *Taenia* spp. samples sequenced were shown to be *T. crassiceps* which is further evidence that domestic dogs are preying on small mammal species. However, there were insufficient numbers of *T. crassiceps* samples to infer any differences in predation behaviour of dogs in different villages. *Taenia crassiceps* cysticercosis has occasionally caused severe disease in humans (Deplazes *et al.*, 2019).

An interesting observation was that in fecal samples only eggs of *E. granulosus* s.l. had significantly higher egg count than samples containing *Taenia* spp. Gemmell (1990) demonstrated that *T. hydatigena* had a substantially higher biotic potential than *E. granulosus* s.l. Dogs infected with *T. hydatigena* producing ~38 000 eggs per day were compared to dogs infected with *E. granulosus* with an estimated 8470 eggs per day. The majority of the *Taenia* spp. samples that were sequenced were shown to be *T. hydatigena*. Thus it could be expected that the intensity of egg contamination of feces of dogs infected with *Taenia* spp. would be far higher than those infected with *E. granulosus* s.l. This anomaly is likely explained by the fact that most *Taenia* spp. eggs remain in the proglottids following expulsion from an infected dog, and the proglottids are either voided separately from the feces or migrate from the feces following defecation by the dog. Deplazes and Eckert (1988*b*) observed that only 36% of the expelled proglottids from dogs experimentally infected with *T. hydatigena* were associated with defecation. Further analyses revealed that only 16% of the total estimated number of eggs were recovered from the feces; the remainder were excreted independently of defecation with the proglottids. Assuming this is the case in the present study, the total number of eggs voided by an infected dog would be over 6 times the numbers detected in fecal samples. This then reconciles the results of the present study to earlier studies demonstrating the much higher biotic potential of *T. hydatigena* compared to *E. granulosus* s.l.

Dogs are the main definitive hosts for *E. granulosus* s.l. Data suggest that, for *E. multilocularis*, they may be an important source of infection for human AE (Torgerson *et al.*, 2020). Consequently, regular treatment of dogs with praziquantel is an important intervention to avoid human disease (World Health Organization, 2011). The efficiency of an intervention programme in which dogs are medicated depends on the number of registered dogs. Furthermore, the acceptance of the community as well as the access to households are decisive points for the efficiency of such an intervention programme. An aggravating factor in the control of these zoonotic diseases is the traditional movement of livestock. The farmers move their animals between the valley and the pastures for 6–8 months per year in order to find rich grazing land. With the livestock the dogs also move each season which increases the possibility of a reinfection (World Bank, 2011).

Although this study found that there is no obvious correlation between the increase in AE (and CE) cases in humans and the contamination with *E. multilocularis* (or *E. granulosus* s.l.) in dog feces in public areas, the role of the dog cannot be excluded given the increase in human AE. To clarify this question conclusively, further parameters need to be examined. The involvement of wild definitive hosts contaminating the environment, changes in the infection cycle and in the pathogenicity of the parasite, the susceptibility of the population as well as changes in environmental factors are only a few parameters that need to be investigated in more detail.

It has been argued that humans are relatively resistant to AE. This might result in the low incidence of human AE in Switzerland despite widespread contamination of the environment with fox feces and hence potential exposure of the population (Gottstein *et al.*, 2015). However, there are subtle suggestions in our results that may provide evidence against this hypothesis. The contamination of the public areas with *E. multilocularis* eggs is similar in the 2 districts; yet there are marked differences in the reported human incidence of disease, although different levels of contamination in private gardens cannot be discounted. Both populations are ethnic Kyrgyz and it would seem unlikely that 1 population would be markedly more resistant to the parasite than the other. Furthermore, the level of contamination with *E. granulosus* s.l. seems to be somewhat higher that of *E. multilocularis* in the Alay districts. Yet in Alay there is a considerably higher incidence of AE than CE, which may point to a greater susceptibility to AE.

## Supporting information

Abdykerimov et al. supplementary material 1 Abdykerimov et al. supplementary material

Abdykerimov et al. supplementary material 2 Abdykerimov et al. supplementary material

Abdykerimov et al. supplementary material 3 Abdykerimov et al. supplementary material

## Data Availability

All data is available in the manuscript or supporting material.

## Supplementary material

The supplementary material for this article can be found at https://doi.org/10.1017/S003118202300118X
