# Supplementary material for: Environmental distribution of Echinococcus- and Taenia spp.-contaminated dog feces in Kyrgyzstan
Source: Parasitology. 2023 Nov 29;151(1):84–92. doi: 10.1017/S003118202300118X (PMC10941041; doi:10.1017/S003118202300118X)
Supplement: Abdykerimov et al. supplementary material 3 — Abdykerimov et al. supplementary material [file S003118202300118Xsup003.pdf]

Supplemental material 3

Maps of villages giving locations of the faecal samples observed and collected.

Maps of villages

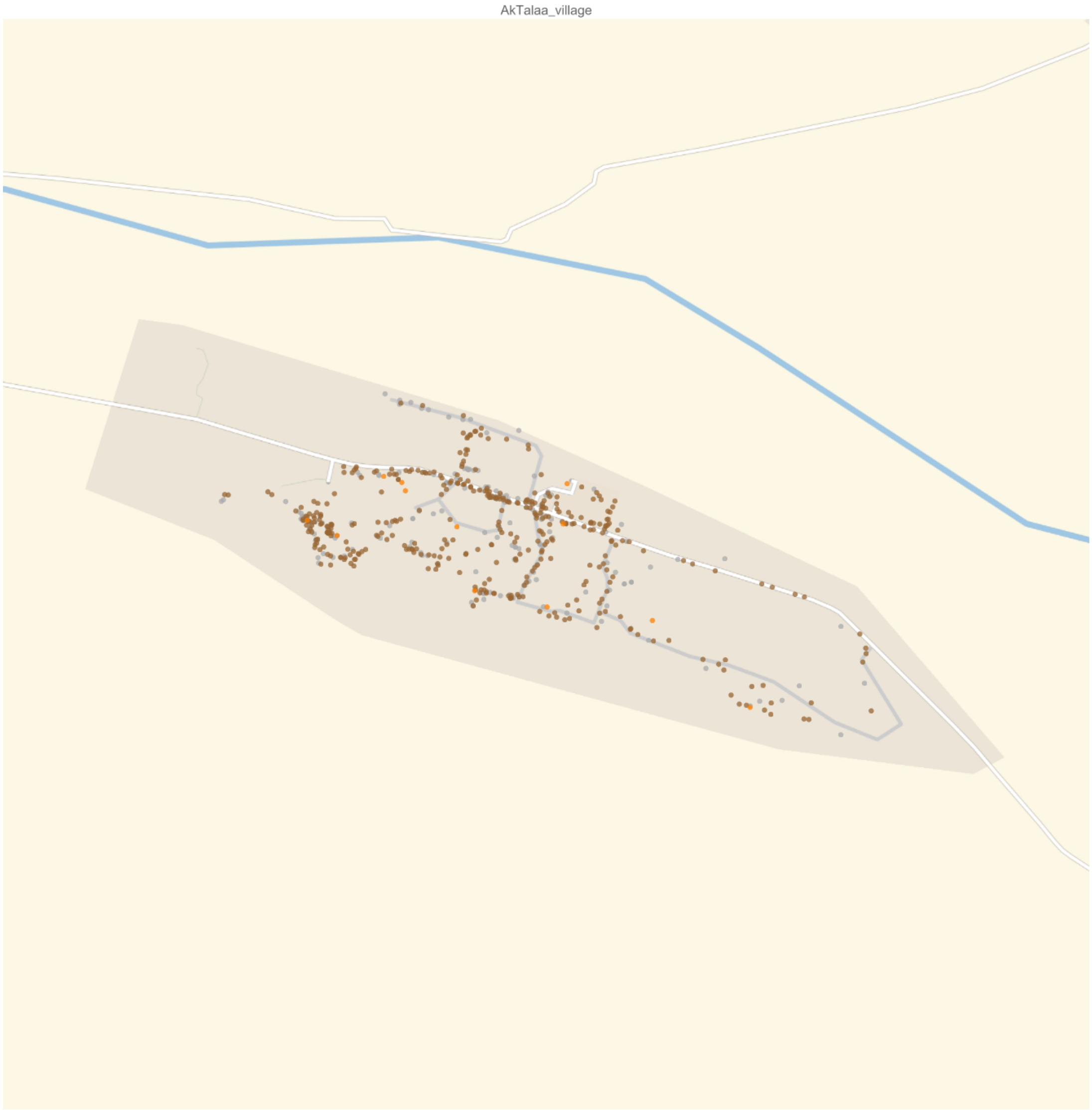

Figure S3 1. Environmental contamination of dog faeces Ak-Talaa village. Position of faecal samples containing eggs of: *E. granulosus* s.l. ●, *E. granulosus s.l.* and *Taenia* spp ●, *E. multilocularis* ●, *E. multilocularis* and *Taenia* spp ●,*Taenia* spp ●, negative ●, old samples ●.

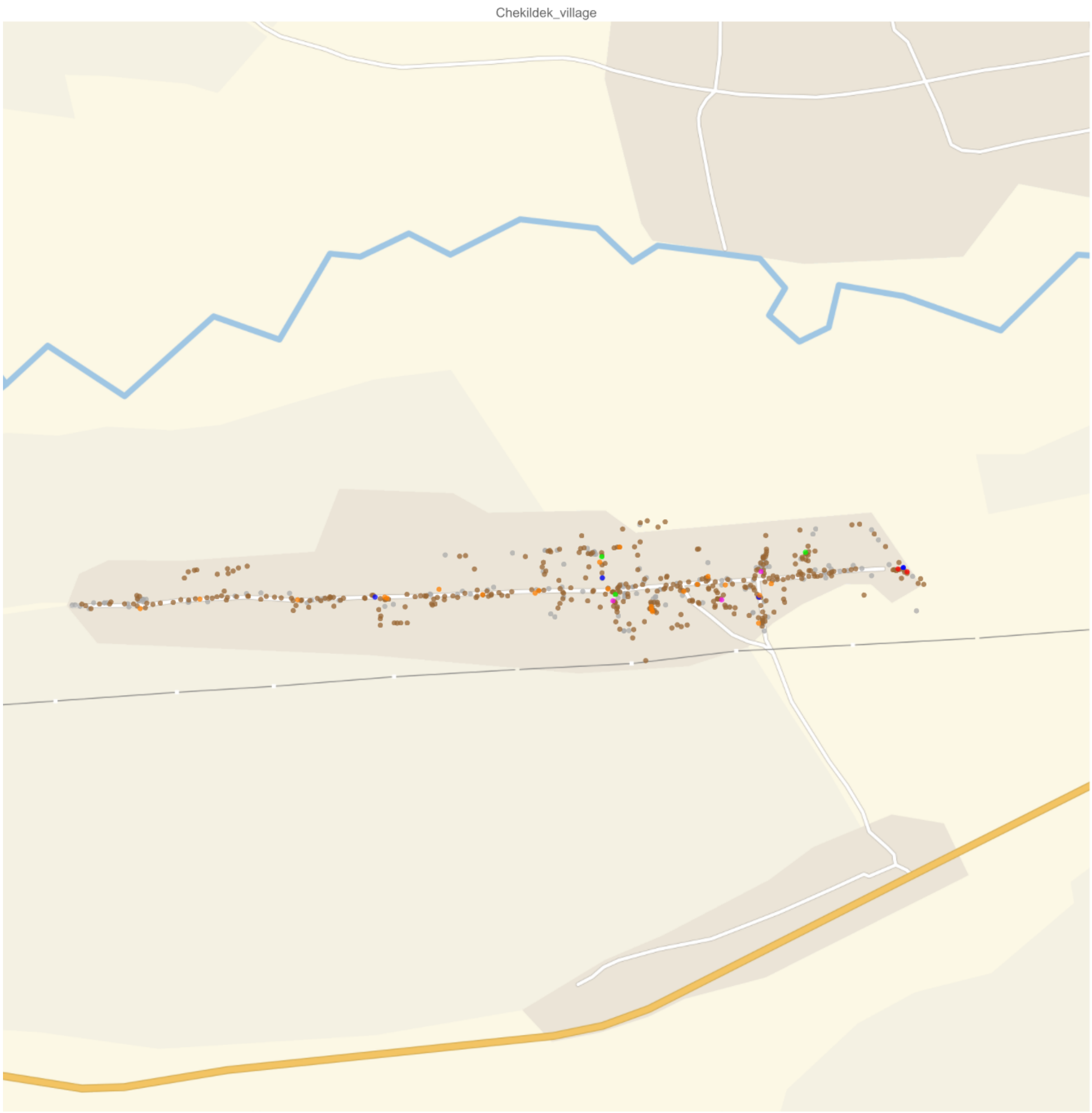

Figure S3 2. Environmental contamination of dog faeces Chekildek village. Position of faecal samples containing eggs of: *E. granulosus* s.l. ●, *E. granulosus s.l.* and *Taenia* spp ●, *E. multilocularis* ●, *E. multilocularis* and *Taenia* spp ●,*Taenia* spp ●, negative ●, old samples ●.

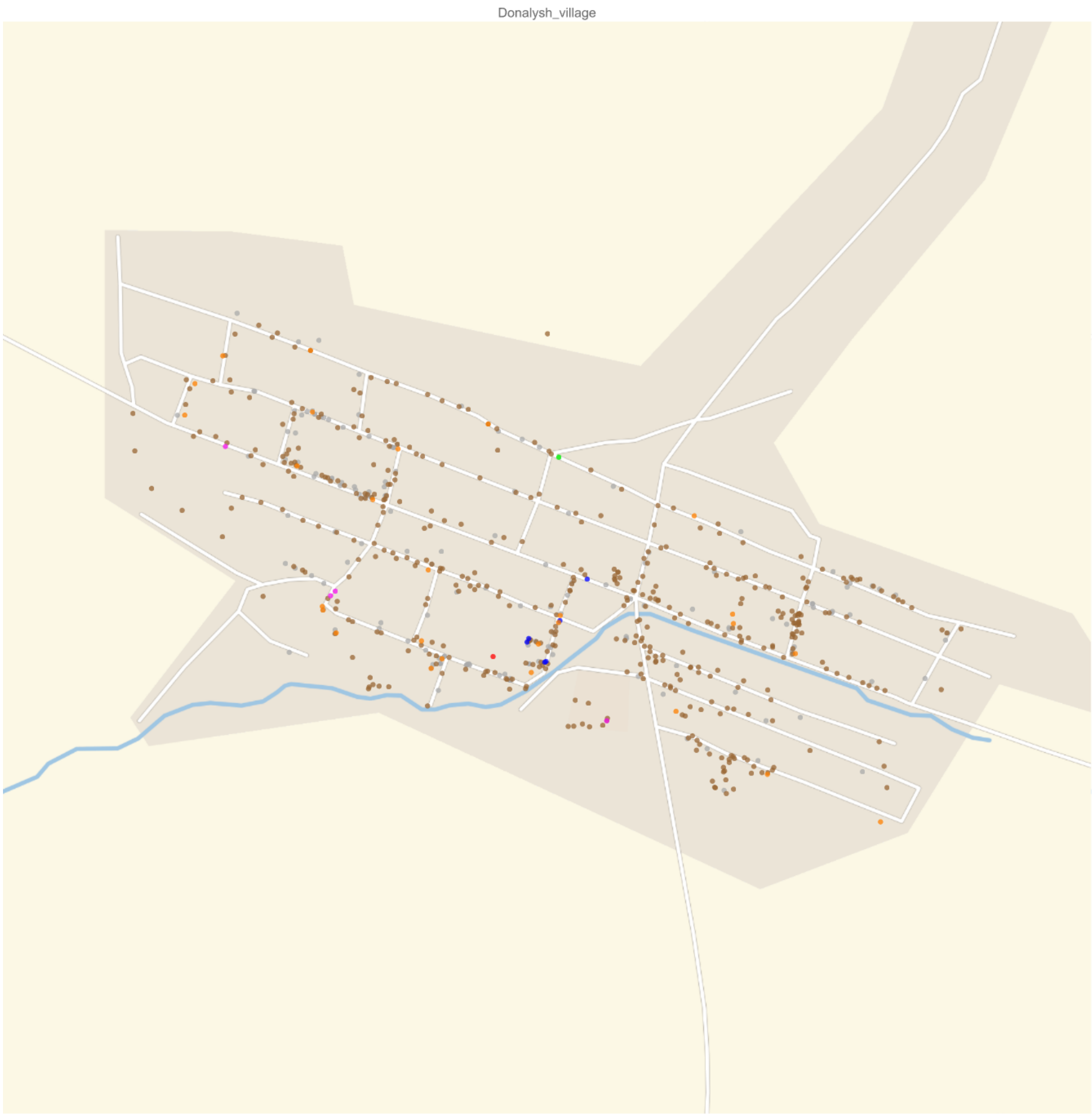

Figure S3 3. Environmental contamination of dog faeces Don-Alysh village. Position of faecal samples containing eggs of: *E. granulosus* s.l. ●, *E. granulosus s.l.* and *Taenia* spp ●, *E. multilocularis* ●, *E. multilocularis* and *Taenia* spp ●, *Taenia* spp ●, negative ●, old samples ●.

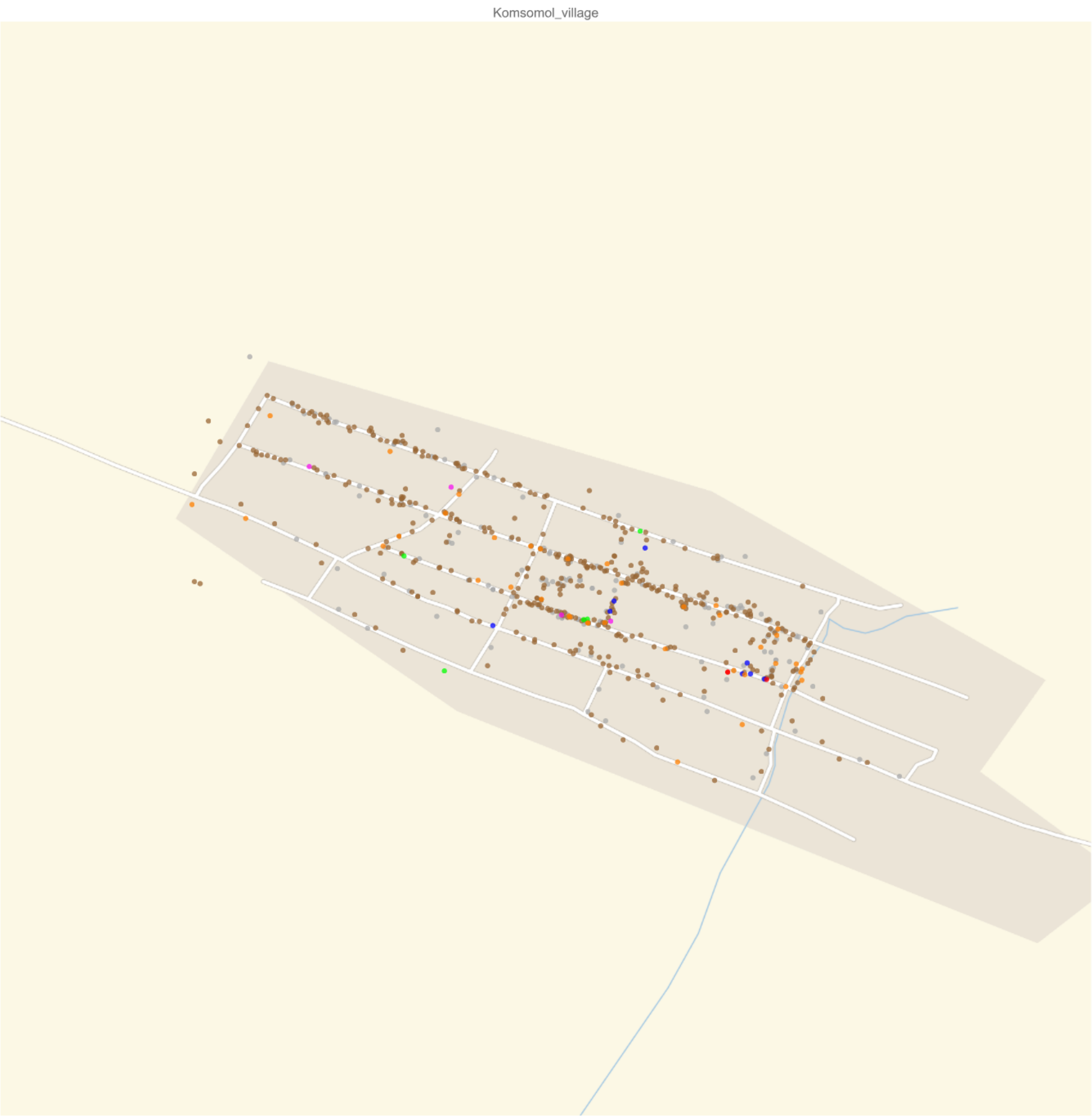

Figure S3 4. Environmental contamination of dog faeces Komsomol village. Position of faecal samples containing eggs of: *E. granulosus* s.l. ●, *E. granulosus s.l.* and *Taenia* spp ●, *E. multilocularis* ●, *E. multilocularis* and *Taenia* spp ●,*Taenia* spp ●, negative ●, old samples ●.

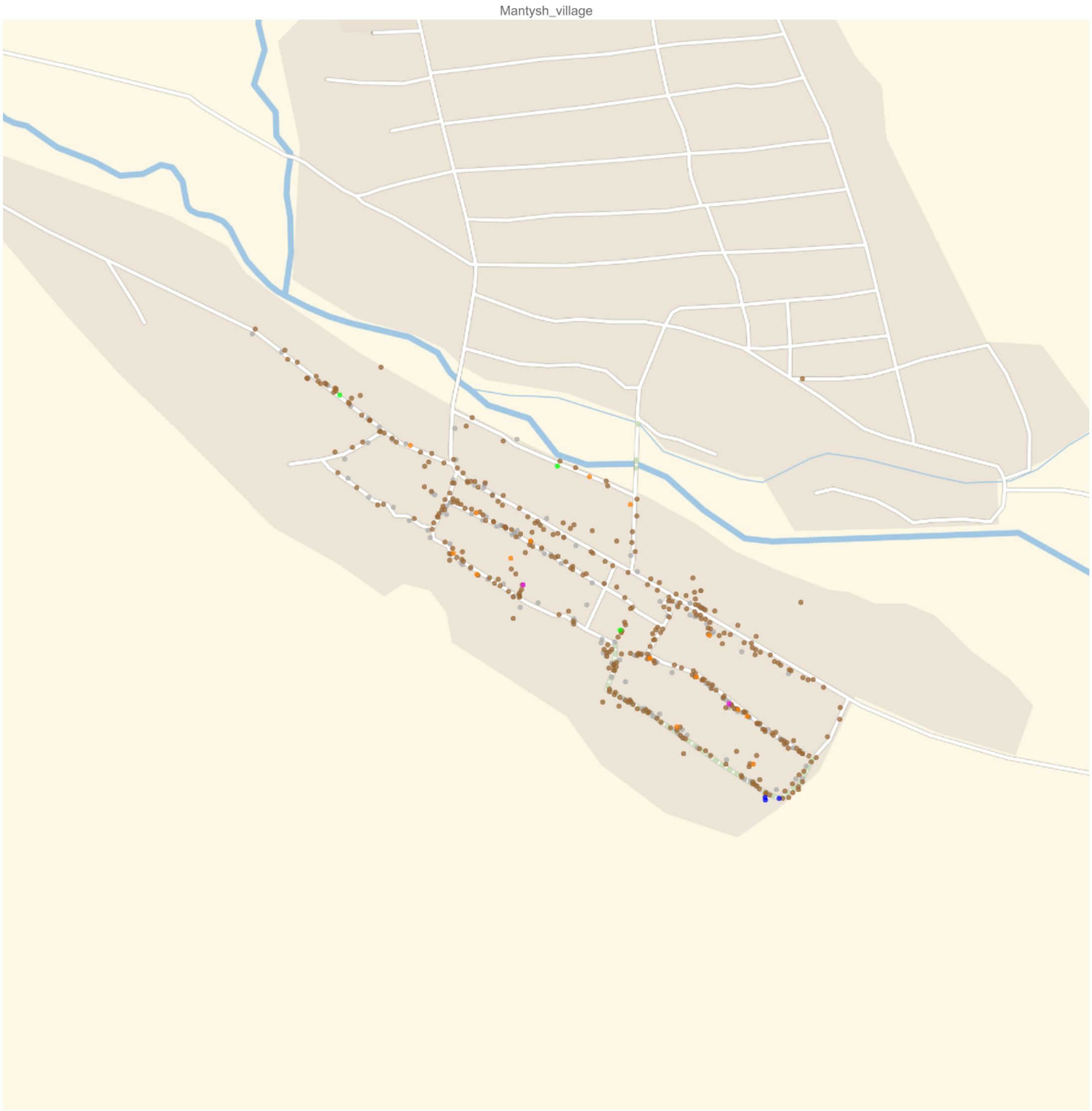

Figure S3 5. Environmental contamination of dog faeces Mantysh village. Position of faecal samples containing eggs of: *E. granulosus* s.l. ●, *E. granulosus s.l.* and *Taenia* spp ●, *E. multilocularis* ●, *E. multilocularis* and *Taenia* spp ●, *Taenia* spp ●, negative ●, old samples ●.

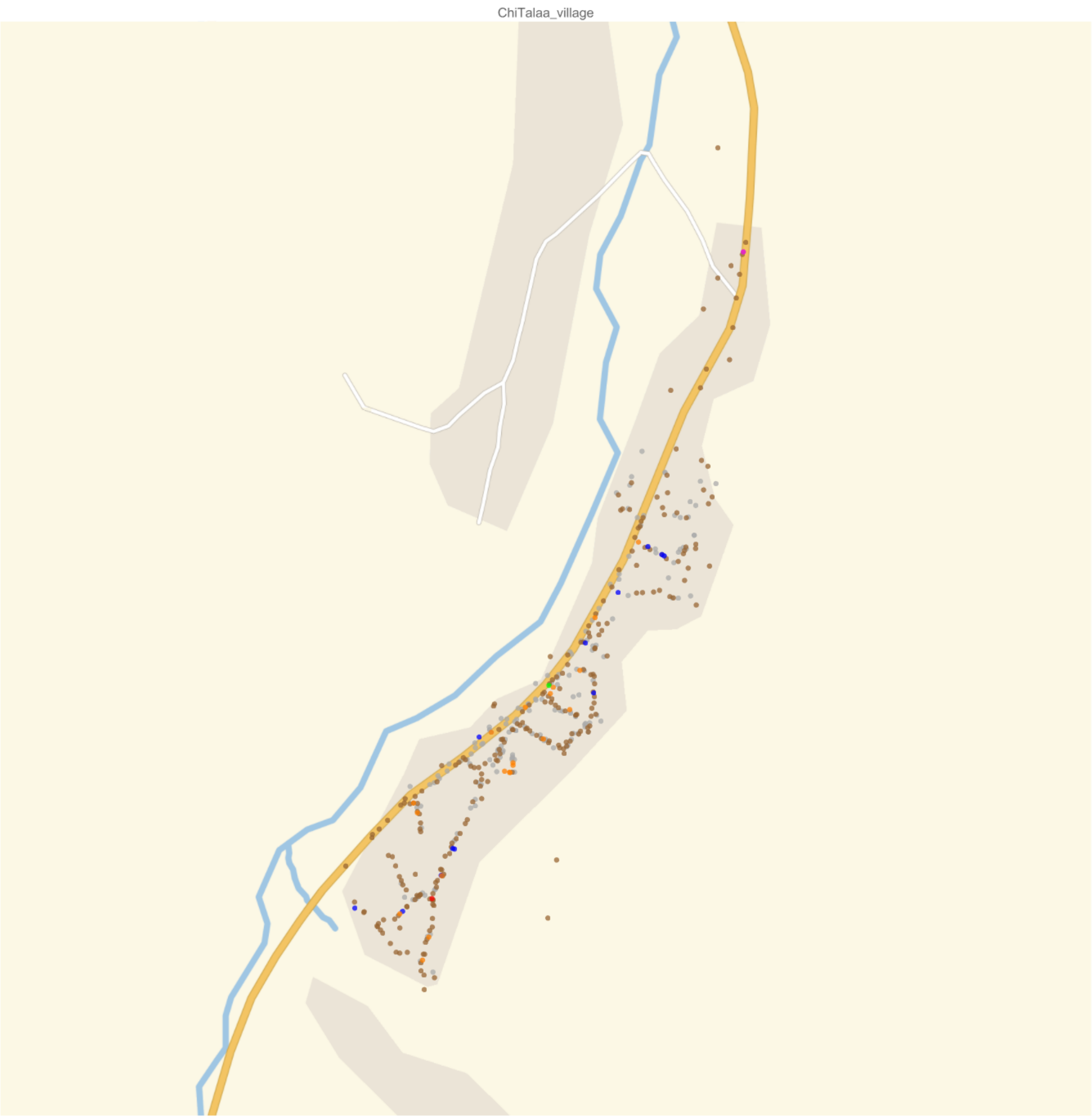

Figure S3 6. Environmental contamination of dog faeces Chii-Talaa village. Position of faecal samples containing eggs of: *E. granulosus* s.l. ●, *E. granulosus s.l.* and *Taenia* spp ●, *E. multilocularis* ●, *E. multilocularis* and *Taenia* spp ●, *Taenia* spp ●, negative ●, old samples ●.

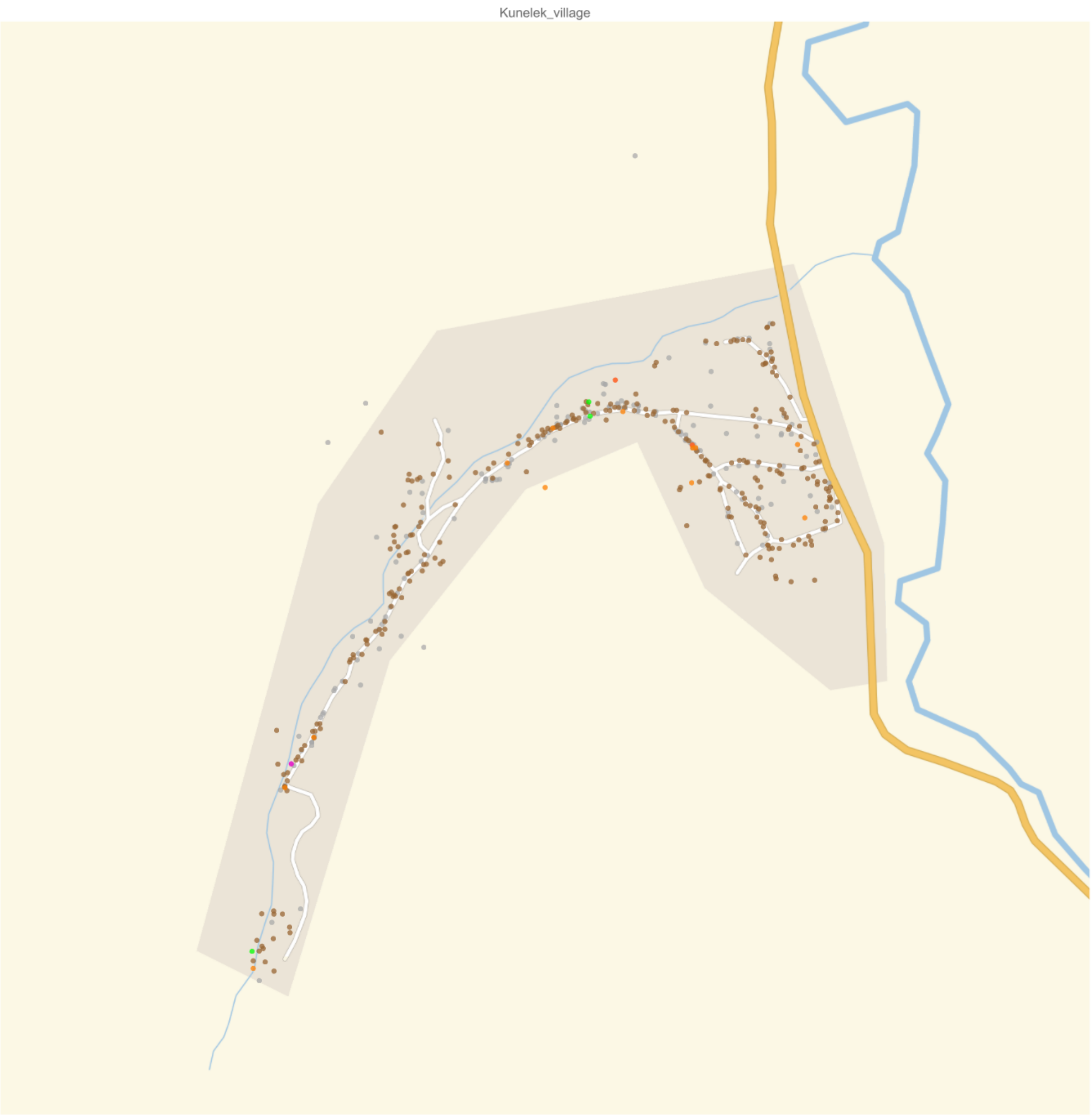

Figure S3 7. Environmental contamination of dog faeces Kun-Elek village. Position of faecal samples containing eggs of: *E. granulosus* s.l. ●, *E. granulosus s.l.* and *Taenia* spp ●, *E. multilocularis* ●, *E. multilocularis* and *Taenia* spp ●, *Taenia* spp ●, negative ●, old samples ●.

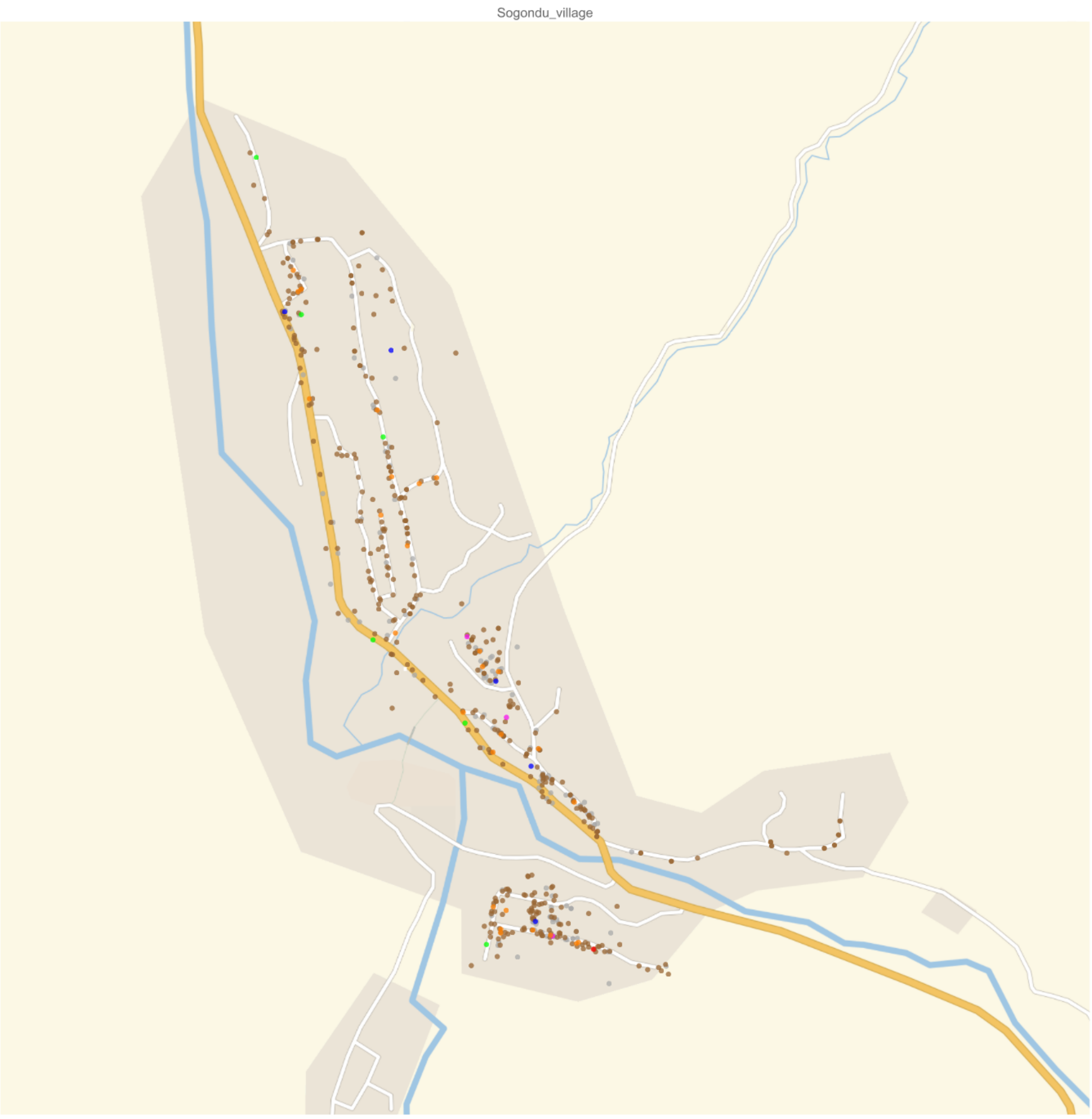

Figure S3 8. Environmental contamination of dog faeces Sogondu village. Position of faecal samples containing eggs of: *E. granulosus* s.l. ●, *E. granulosus s.l.* and *Taenia* spp ●, *E. multilocularis* ●, *E. multilocularis* and *Taenia* spp ●, *Taenia* spp ●, negative ●, old samples ●.

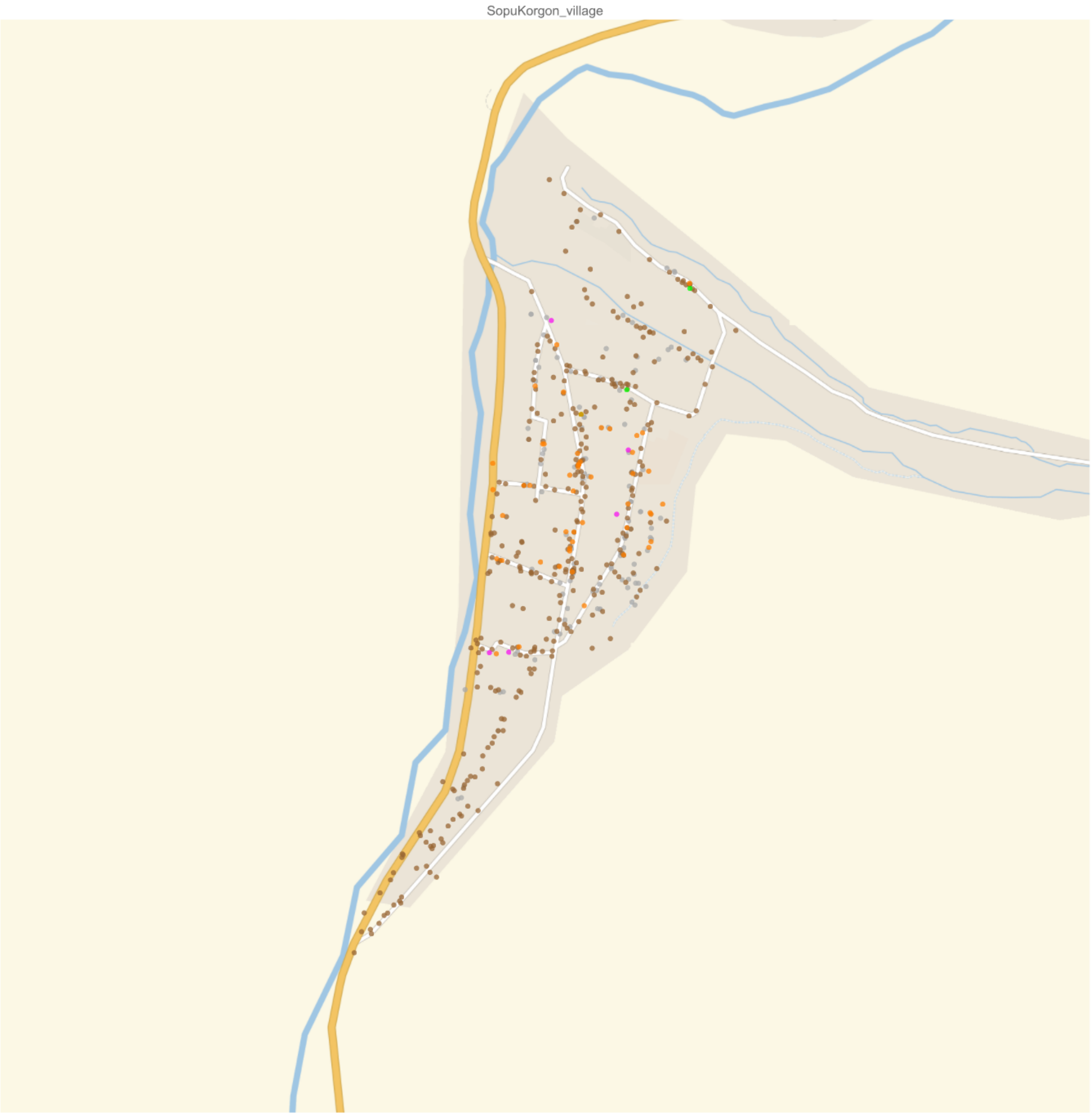

Figure S3 9. Environmental contamination of dog faeces Sopu-Korgon village. Position of faecal samples containing eggs of: *E. granulosus* s.l. ●, *E. granulosus s.l.* and *Taenia* spp ●, *E. multilocularis* ●, *E. multilocularis* and *Taenia* spp ●, *Taenia* spp ●, negative ●, old samples ●.

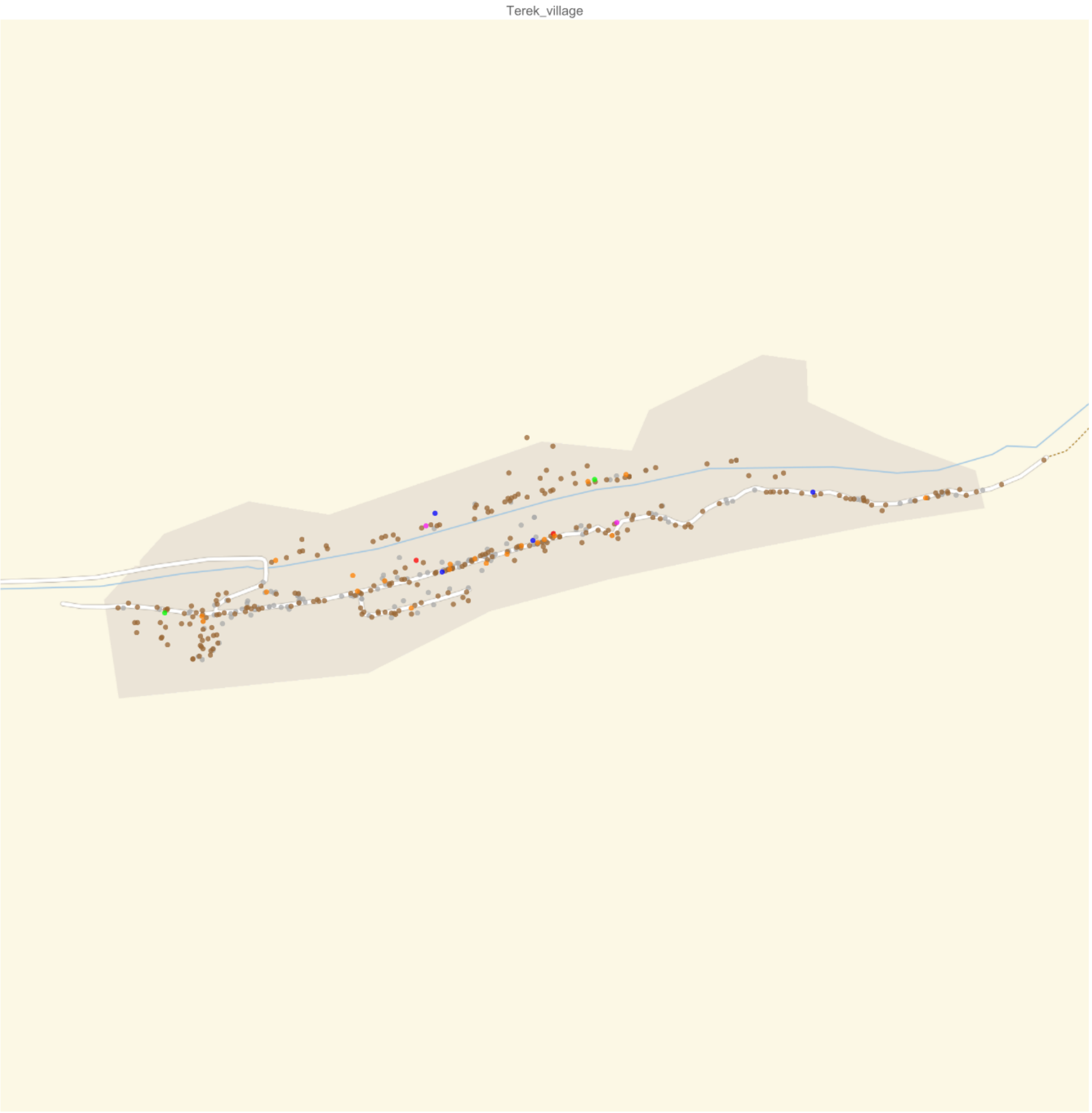

Figure S3 10. Environmental contamination of dog faeces Terek village. Position of faecal samples containing eggs of: *E. granulosus* s.l. ●, *E. granulosus s.l.* and *Taenia* spp ●, *E. multilocularis* ●, *E. multilocularis* and *Taenia* spp ●, *Taenia* spp ●, negative ●, old samples ●.
